# Risk factors and outcomes for the Q151M and T69 insertion HIV-1 resistance mutations in historic UK data

**DOI:** 10.1186/s12981-018-0198-7

**Published:** 2018-04-16

**Authors:** Oliver T. Stirrup, David T. Dunn, Anna Tostevin, Caroline A. Sabin, Anton Pozniak, David Asboe, Alison Cox, Chloe Orkin, Fabiola Martin, Patricia Cane, the UK HIV Drug Resistance Database, David Asboe, Anton Pozniak, Patricia Cane, David Chadwick, Duncan Churchill, Duncan Clark, Simon Collins, Valerie Delpech, Samuel Douthwaite, David Dunn, Esther Fearnhill, Kholoud Porter, Anna Tostevin, Oliver Stirrup, Christophe Fraser, Anna Maria Geretti, Rory Gunson, Antony Hale, Stéphane Hué, Steve Kaye, Linda Lazarus, Andrew Leigh-Brown, Tamyo Mbisa, Nicola Mackie, Samuel Moses, Chloe Orkin, Eleni Nastouli, Deenan Pillay, Andrew Phillips, Caroline Sabin, Erasmus Smit, Kate Templeton, Peter Tilston, Ian Williams, Hongyi Zhang, Keith Fairbrother, Jane Greatorex, Siobhan Oâ€™Shea, Jane Mullen, Alison Cox, Richard Tandy, Tracy Fawcett, Mark Hopkins, Clare Booth, Ana Garcia-Diaz, Lynne Renwick, Matthias L. Schmid, Brendan Payne, Jonathan Hubb, Stuart Kirk, Amanda Bradley-Stewart, the UK Collaborative HIV Cohort, Jonathan Ainsworth, Sris Allan, Jane Anderson, Abdel Babiker, David Chadwick, Duncan Churchill, Valerie Delpech, David Dunn, Brian Gazzard, Richard Gilson, Mark Gompels, Phillip Hay, Teresa Hill, Margaret Johnson, Sophie Jose, Stephen Kegg, Clifford Leen, Fabiola Martin, Dushyant Mital, Mark Nelson, Chloe Orkin, Adrian Palfreeman, Andrew Phillips, Deenan Pillay, Frank Post, Jillian Pritchard, Caroline Sabin, Achim Schwenk, Anjum Tariq, Roy Trevelion, Andy Ustianowski, John Walsh, Alicia Thornton, Susie Huntington, Adam Glabay, Shaadi Shidfar, Janet Lynch, James Hand, Carl de Souza, Nicky Perry, Stuart Tilbury, Elaney Youssef, Tracey Mabika, David Asboe, Sundhiya Mandalia, Sajid Munshi, Ade Adefisan, Chris Taylor, Zachary Gleisner, Fowzia Ibrahim, Lucy Campbell, Kirsty Baillie, Nataliya Brima, Ian Williams, Sheila Miller, Chris Wood, Mike Youle, Fiona Lampe, Colette Smith, Rob Tsintas, Clinton Chaloner, Samantha Hutchinson, Nicky Mackie, Alan Winston, Jonathan Weber, Farhan Ramzan, Mark Carder, Alan Wilson, Sheila Morris, Sue Allan, Adam Lewszuk, Akin Faleye, Victoria Ogunbiyi, Sue Mitchell, Christian Kemble, Sarah Russell-Sharpe, Janet Gravely, Andrew Harte, Hazel Spencer, Ron Jones, Shirley Cumming, Claire Atkinson, Veronica Edgell, Julie Allen, Cynthia Murphy, Ilise Gunder

**Affiliations:** 1 Centre for Clinical Research in Infection and Sexual Health, Institute for Global Health, University College London, London, UK; 2 Centre for Clinical Research, Epidemiology, Modelling and Evaluation, Institute for Global Health, University College London, Royal Free Campus, London, UK; 3 Chelsea and Westminster Hospital NHS Foundation Trust, London, UK; 4 Infection and Immunity laboratory, Charing Cross Hospital, London, UK; 5 Barts Health NHS Trust, London, UK; 6 Hull York Medical School, University of York, York, UK; 7 Faculty of Medicine, University of Queensland, Brisbane, Australia; 8 High Containment Microbiology, Public Health England, Salisbury, UK

**Keywords:** 151 complex, 69 insertion complex, HIV, Multidrug resistance, Multi-NRTI resistance, NRTI

## Abstract

**Background:**

The prevalence of HIV-1 resistance to antiretroviral therapies (ART) has declined in high-income countries over recent years, but drug resistance remains a substantial concern in many low and middle-income countries. The Q151M and T69 insertion (T69i) resistance mutations in the viral reverse transcriptase gene can reduce susceptibility to all nucleoside/tide analogue reverse transcriptase inhibitors, motivating the present study to investigate the risk factors and outcomes associated with these mutations.

**Methods:**

We considered all data in the UK HIV Drug Resistance Database for blood samples obtained in the period 1997–2014. Where available, treatment history and patient outcomes were obtained through linkage to the UK Collaborative HIV Cohort study. A matched case–control approach was used to assess risk factors associated with the appearance of each of the mutations in ART-experienced patients, and survival analysis was used to investigate factors associated with viral suppression. A further analysis using matched controls was performed to investigate the impact of each mutation on survival.

**Results:**

A total of 180 patients with Q151M mutation and 85 with T69i mutation were identified, almost entirely from before 2006. Occurrence of both the Q151M and T69i mutations was strongly associated with cumulative period of virological failure while on ART, and for Q151M there was a particular positive association with use of stavudine and negative association with use of boosted-protease inhibitors. Subsequent viral suppression was negatively associated with viral load at sequencing for both mutations, and for Q151M we found a negative association with didanosine use but a positive association with boosted-protease inhibitor use. The results obtained in these analyses were also consistent with potentially large associations with other drugs. Analyses were inconclusive regarding associations between the mutations and mortality, but mortality was high for patients with low CD4 at detection.

**Conclusions:**

The Q151M and T69i resistance mutations are now very rare in the UK. Our results suggest that good outcomes are possible for people with these mutations. However, in this historic sample, viral load and CD4 at detection were important factors in determining prognosis.

**Electronic supplementary material:**

The online version of this article (10.1186/s12981-018-0198-7) contains supplementary material, which is available to authorized users.

## Background

Highly potent and effective antiretroviral therapies (ART) to treat HIV-1 infection are now available and although drug resistance was a considerable problem in the early years of ART use, its impact has now declined in high-income countries. In the UK HIV Drug Resistance Database (UK-HDRD) only about 30% of treated patients receiving resistance tests following virological failure in 2014 showed any drug resistance, compared with 72% in 2002 [1]. Likewise, a decline in drug resistance in treated patients has been observed in Canada [2] and overall in Western Europe [3]. In Switzerland it is now considered that the emergence of new drug resistance “can be virtually stopped with new potent therapies and close monitoring” [4]. However, drug resistance remains a considerable problem in the successful treatment of HIV infection in many low- and middle-income countries (LMICs) [5–7]. This is in part due to limited drug options, but also to a lack of (or limited) routine viral load (VL) monitoring [8] as patients continuing to use ART while failing to suppress viral replication may develop resistance mutations. This has led to calls for improved provision of VL monitoring and resistance testing [9–11].

HIV mutations that confer resistance to multiple drugs, and hence have the potential to severely limit treatment options, are of particular concern. The T69 insertion (T69i) and associated mutations in reverse transcriptase confer high level resistance to all nucleoside/nucleotide reverse transcriptase inhibitors (NRTIs) [12]. Likewise the Q151M mutation causes intermediate/high-level resistance to zidovudine (ZDV), didanosine (DDI), stavudine (D4T), and abacavir (ABC) and low level resistance to tenofovir (TDF), lamivudine (3TC) and emtricitabine (FTC) [12]. The Stanford University Drug Resistance Database reports that in combination with mutations at associated positions (62, 75, 77 and 116) Q151M confers high-level resistance to ZDV, DDI, D4T and ABC, and intermediate resistance to TDF, 3TC and FTC [13].

The T69i mutation has been linked to DDI use [14] whilst for Q151M an association with D4T has been observed [15], and most reports of the mutations occurring in Europe date to 10 or more years ago when the use of these drugs was still widespread [14, 16, 17]. However, a relatively high prevalence of Q151M has been observed in more recent studies of patients failing ART from LMICs [15, 18–23] (ranging 2–14% in these studies). Whilst a fall in prevalence is to be expected to follow from the complete phase-out of D4T use [24], there is the potential for cases of the mutation to persist for years to come. Although the occurrence of the T69i mutation appears to remain rare even in LMICs (ranging 0–1% in these studies [15, 18–22], with the 1% value based on a single patient in Saravanan et al. [21]), there nonetheless remains a motivation to investigate outcomes in affected patients given the potential impact on future treatment. Some recent studies have found that the presence of NRTI resistance is actually predictive of successful virological suppression on second-line ART [25, 26], so it is of interest to evaluate whether boosted protease inhibitor regimens are effective in those cases with these specific rare mutations.

This paper reports changes in the prevalence of multi-drug resistance mutations Q151M and T69i in the UK-HDRD over time and evaluates outcomes in affected patients. Risk factors for the occurrence of the mutations and predictors of subsequent viral suppression are investigated, with the aim of helping to inform clinical management in regions in which the development of drug resistance is an increasing concern.

## Methods

### Data and general approach

We considered all available data in the UK-HDRD for blood samples obtained in the period 1997–2014. Few data are available before this period, as resistance testing was not widely available, with no recorded observations of either Q151M or T69i mutations. The prevalence of each resistance mutation studied was assessed in relation to calendar time stratified by whether patients were recorded as ART-naïve or -experienced in the UK-HDRD. Where possible, clinical data were obtained through linkage to the UK Collaborative HIV Cohort (UK CHIC) study [27]. Patients without classification recorded were assumed to be ART-experienced for the evaluation of prevalence, but ART-status was available in all UK CHIC-linked patients for the analyses of risk factors and outcomes.

The identification of drug resistance mutations was based on output from the Stanford University HIV Drug Resistance Database program. Following established convention (and software output) [13], the term ‘T69i mutation’ is used throughout to refer to any insertion in the β3–β4 loop of reverse transcriptase between codons 66 and 70.

A Bayesian approach to statistical analysis was used throughout, with models implemented in the Stan probabilistic programming language [28] using the rstan [29] interface for R. This approach was chosen to guard against the erroneous inferences that can arise from model building based on large numbers of sequential classical hypothesis tests [30]. Continuous variables were standardised for analyses (by subtracting their mean and then dividing by their SD) so that their associated parameters are on a comparable scale. Additional technical details are provided in Additional file 1.

### Risk factors for development of mutations

Matched case–control analyses were conducted in order to investigate factors associated with the occurrence of each of the mutations studied. These analyses only included patients for whom resistance test data could be matched to clinical records in the UK CHIC study. ART-experienced patients were defined as ‘cases’ at time of blood sampling for the first observation of the relevant mutation and were matched in a 1:10 ratio to ‘controls’, who were randomly sampled (without replacement) from a subset of patients with at least one resistance test available for whom the relevant mutation had never been detected. Matching in each instance was conditional on the control patient having first initiated ART within 6 months (in calendar time) of the case patient doing so, and current and historic treatment variables for both case and control patients were defined with respect to the time of blood sampling for the resistance test in the case patient. Multivariable conditional logistic regression analyses were conducted accounting for the matched case–control groups. This form of analysis was chosen so that key factors associated with the development of the resistance mutations could be investigated whilst controlling for the evolution of ART drug combinations and treatment strategies over time. The complexity of ART histories for each patient and the limited numbers of cases available for analysis precluded more complex modelling of the probability of the development of each resistance mutation conditional on full details of ART history.

Details of variable selection for these models are provided in Additional file 1. For some patients included in the UK-HDRD, the original viral sequence data is not recorded and so it is not possible to obtain a viral subtype. As such, viral subtype was added to the final model in the subset of patients for whom this was possible (using REGA, classified as A, B, C, circulating recombinant form (CRF), or other).

### Factors associated with successful viral suppression following detection of resistance

Multivariable Cox regression was used to investigate the factors associated with successful viral suppression following detection of either the Q151M or T69i mutation. Change to ART regimen following detection of a resistance mutation was taken as the zero time point for these analyses, and the survival outcome was defined as the time to the first of two consecutive undetectable VL observations (considering observations < 200 copies/mL to be ‘undetectable’). Patients in whom there was no change to ART regimen recorded were not included in these analyses. Baseline CD4 cell counts and VL were defined as the most recent observation recorded within 6 months prior to the start of the new ART regimen, and outcomes were censored at any subsequent change to ART regimen.

In the Cox regression analyses, baseline square-root(CD4) and log_10_(VL) were included as predictors, alongside treatment-naïve or experienced status, sex, patient age and variables representing individual drugs received by ≥ 10% patients. Protease inhibitors (PIs) were grouped into the presence or absence of un-boosted or ritonavir-boosted PI (ubPI/rbPI) and non-nucleoside/nucleotide reverse transcriptase inhibitors (NNRTIs) were also grouped into a single variable. A dummy variable was included to indicate patients receiving an ART regimen containing drugs from only a single class. Presence or absence of the K65R mutation was also included as a predictor, and analyses were also conducted including the respective accessory mutations for Q151M and T69i. Further analyses were conducted to investigate the durability of viral suppression in ART-experienced patients, based on the proposal of McKinnon et al. [31]; briefly, viral suppression and subsequent virological failure are modelled using sequential Weibull time-to-event models so that the proportion of patients with sustained viral suppression can be estimated (Additional file 1).

### Factors associated with mortality

To investigate the association between Q151M and 69i and mortality, we carried out a matched cohort analysis of comparable patients in whom ART data were available. Patients were matched from a subset of patients with at least one resistance test available for whom the relevant mutation had never been detected. Matching was conditional on the observation of a resistance test within 12 months of that in the case patient (effectively matching on treatment failure or interruption in ART-experienced patients), date of ART initiation within 12 months of the case patient in those who were ART-experienced at the time of detection (or ART-naïve status in ART-naïve cases), and patient sex and mode of infection. Matching was carried out with up to 10 ‘controls’ for each ‘case’ (dependent on availability). Survival analyses (for the outcome of any-cause death from date of resistance test) were then conducted using Cox regression including a normally distributed shared frailty term for each matched group and adjusted for CD4 count at time of resistance test, age at resistance test, prior diagnosis of an AIDS-defining illness at resistance test and number of other reverse transcriptase resistance mutations detected at the index resistance test (grouping ≥ 9 mutations).

## Results

### Q151M

**Fig. 1:**
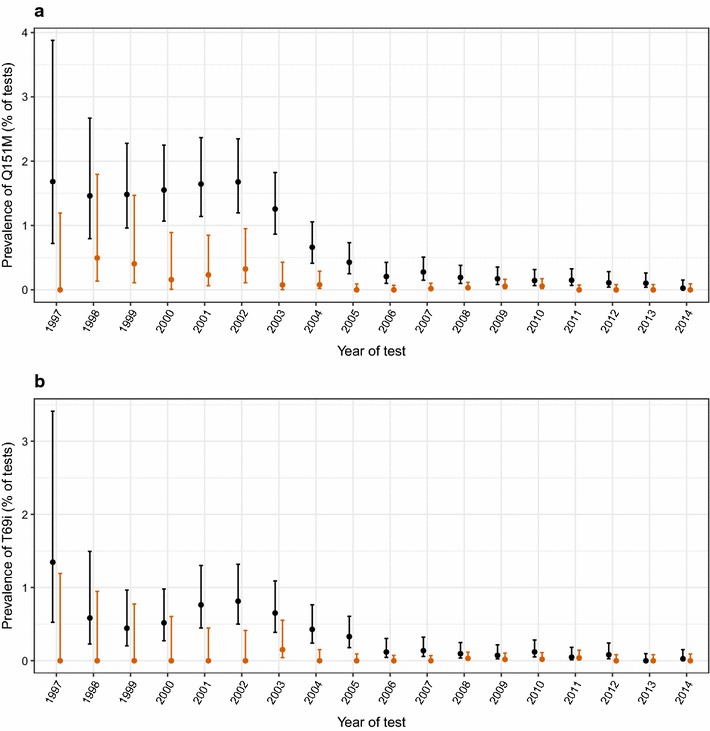
Prevalence of (**a**) the Q151M mutation and (**b**) the T69i mutation per patient by calendar year of sequencing (patients can be included in multiple calendar years, but are only counted once per year), according to whether the patient was ART experienced (black circle) or naïve (orange circle) at the time of blood sample. The denominator in each year is the total number of patients with at least one reverse transcriptase sequence recorded in that year. Binomial 95% CIs are shown

A total of 180 patients with at least one observation of the Q151M mutation were recorded in the UK-HDRD, out of a total of 80,281 patients with at least one resistance test. At first observation of the Q151M mutation, 18 (10%) patients were recorded as being ART-naïve, 139 (77%) as experienced and 23 (13%) as ‘not classified’. Figure 1a shows the prevalence of the Q151M mutation by calendar year according to whether the patient was ART experienced or naïve at the time of blood sample. The prevalence of the mutation was consistently low in ART-naïve patients, with a maximum of 0.5% (2/402) of patients in 1998, whilst in ART-experienced patients there was a fall in prevalence from 1.7% (33/1966) in 2002 to 0.2% (8/3376) 2006 and then to 0.027% (1/3733) in 2014.

Table 1 provides a summary of the associated major reverse transcriptase mutations at first observation of the Q151M mutation in each of the affected patients. Only 3% of patients showed no other mutation, and the most common associated major reverse transcriptase mutations were M184V (47%), K103N (35%) and K65R (29%). Data regarding accessory mutations to Q151M [12, 13] were available in 155 (86%) patients: A62V was present in 49 (32%), V75I in 62 (40%), F77L in 47 (30%) and F116Y in 99 (64%), and 75% of patients showed at least one accessory mutation.

**Table 1:** Summary table for associated mutations for Q151M (*n *= 180 patients)

	*n* (%)
Q151M isolated major RT mutation	5 (3)
Q151M accessory mutations^a^	
0	38 (25)
1	50 (32)
2	14 (9)
3	33 (21)
4	20 (13)
NRTI major mutations other than Q151M	
0	20 (11)
1	44 (24)
2	45 (25)
3	32 (18)
4	24 (13)
5	14 (8)
6	1 (1)
TAMs present^b^	
0	82 (46)
1	40 (22)
2	18 (10)
3	23 (13)
4	15 (8)
5	2 (1)
NNRTI major mutations	
0	35 (19)
1	43 (24)
2	64 (36)
3	24 (13)
4	11 (6)
5	3 (2)
PI major mutations^c^	
0	108 (60)
1	33 (18)
2	14 (8)
3	10 (6)
4	9 (5)
5	1 (1)
6	4 (2)
Number of classes with resistance^d^	
1	17 (9)
2	110 (61)
3	53 (29)

^a^Including A62V, V75I, F77L and F116Y, data available for 155 patients

^b^M41L, D67N, K70R, L210W, T215Y/F and K219Q/E

^c^PI PCR failed in one case.

^d^Of NRTI, NNRTI and PI. A full list of mutations and further information regarding associated K65R mutations are provided in Additional file 1

**Fig. 2:**
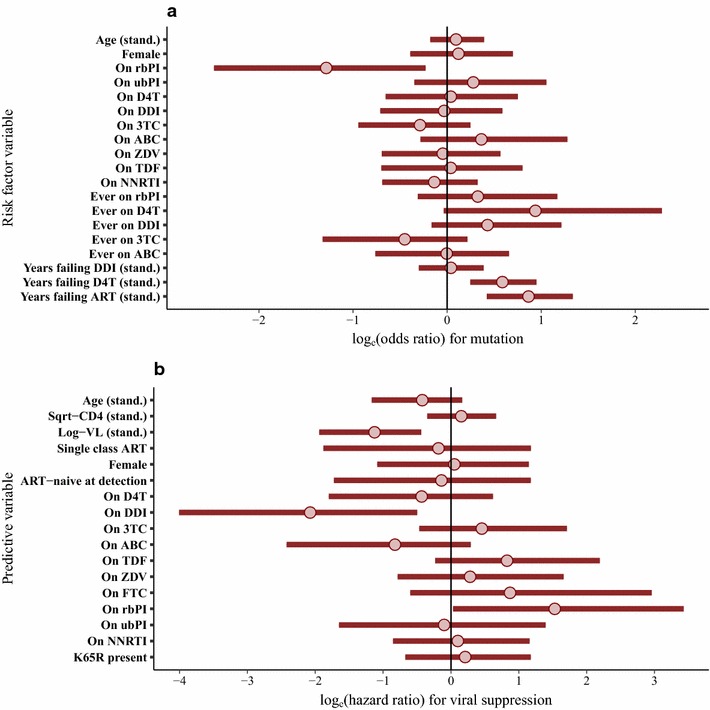
Posterior mean values and 95% credibility intervals for (**a**) log-odds ratios in the matched case–control analysis investigating factors associated with the occurrence of the Q151M mutation and (**b**) log-hazard ratios in the Cox regression for confirmed viral suppression following treatment change after detection of Q151M mutation. Continuous variables were standardised (stand.), by subtracting the mean and dividing by SD, for these analyses. The results presented are from multivariable models in each case

Of the patients in whom the Q151M mutation was detected, 96 could be linked to clinical data. Of these patients, 82 (85%) were recorded as ART-experienced and one as ‘not classified’, with 13 being ART-naïve. Information regarding the ART regimen prior to the resistance test was available in 74/82 (90.2%) of the ART-experienced patients, and these 74 patients were included in the matched case–control analysis to investigate factors associated with the occurrence of the mutation (Fig. 2a). For the final fitted model, the strongest evidence for a positive association was for ‘total years of virological failure’ (standardised odds ratio (sOR), 95% credibility interval (CrI) 2.39, 1.52–3.82; OR of 2.92 on original scale (years)), followed by ‘years of virological failure on D4T’ (sOR 1.80, 1.28–2.59; OR of 1.68 on original scale), whilst ‘current’ treatment using a rbPI showed a negative association (OR 0.28, 0.08–0.79) with occurrence of the mutation (Fig. 2a). There was not strong evidence of any positive or negative association with viral subtype (results in Additional file 1).

In 62/96 (65%) patients, a confirmed undetectable VL was observed (at any point) following the detection of the Q151M mutation at a median of 1.0 (IQR 0.5–2.3) years from the date of the resistance test sample. For a total of 62 patients there were data on change to ART regimen after detection of the Q151M mutation and before viral re-suppression (for patients in whom this occurred), but six of these patients were missing a baseline CD4 cell count (three were also missing baseline VL) and so were dropped from the Cox analysis. Hence, 56 patients were included in the Cox analysis, with a total of 22 events (confirmed viral suppression) observed before censoring. Higher baseline VL (prior to new ART regimen) showed a substantial negative association with the probability of viral suppression (standardised hazard ratio (sHR) 0.32, 0.14–0.64; HR of 0.30 on original scale of log_10_ copies/mL) as did use of DDI (21/56 patients; HR 0.12, 0.02–0.61), whilst the use of a rbPI showed a positive association (31/57 patients; HR 4.62, 1.03–30.88) (Fig. 2b). The model also indicated that TDF use is likely to be associated with suppression, although this result is not definitive. Information regarding the presence or absence of accessory mutations was available for 46 of these patients, but no strong evidence of an association with success of viral suppression was found. An additional analysis was conducted accounting for resistance to PI drugs used in rbPI regimens, with no notable change in the results obtained and no detected reduction in viral suppression when PI resistance was present (further details in Additional file 1).

**Fig. 3:**
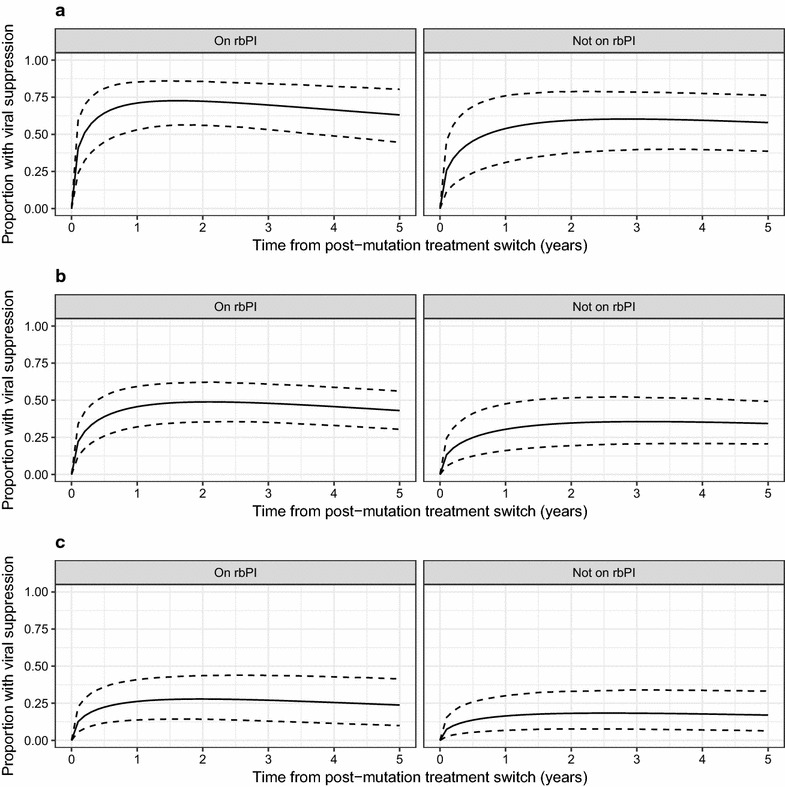
Modelled probability of viral suppression in ART-experienced patients in terms of time since treatment switch following detection of the Q151M mutation for patients with a baseline viral load of (**a**) 2000 copies/mL (≈ 10th centile), (**b**) 40,000 copies/mL (≈ 50th centile) or (**c**) 500,000 copies/mL (≈ 90th centile). Response is modelled according to presence or absence of a ritonavir-boosted protease inhibitor in the ART regimen at time zero, but patients were not censored at change to drug regimen in this analysis. The expected probability (solid line) and 95% credibility interval (dashed lines) from Bayesian fitting of sequential Weibull models for viral suppression and rebound are shown. DDI use was adjusted for in this analysis, but results are shown for patients not on DDI

A model was fitted to assess the durability of viral suppression that included parameters for baseline VL, rbPI and DDI use. This analysis indicated that the durability of viral suppression was good for patients with low baseline VL, but that the overall probability of suppression was worse for patients with higher baseline VL values (Fig. 3).

For the matched cohort analysis of mortality, 72 patients with and 665 patients without the Q151M mutation were included. Higher age and lower CD4 count were strongly associated with mortality, but no clear association with the number of reverse transcriptase resistance mutations was observed. There was no evidence that presence of the Q151M mutation was associated with increased mortality (HR 1.25, 95% CrI 0.84–2.1). In patients with Q151M, most deaths occurred amongst those with CD4 count < 100 cells/µL (12 deaths/24 patients vs 6/54 for patients with CD4 ≥ 100 cells/µL, further details in Additional file 1).

### T69i

A total of 85 patients with at least one detection of the T69i mutation are present in the UK-HDRD. At first observation of the T69i mutation, seven (8.2%) patients were recorded as being ART-naïve, 69 (81.2%) as experienced and nine (10.6%) as ‘not classified’. Figure 1b shows the prevalence of the T69i mutation per patient by calendar year according to whether the patient was ART experienced or naïve at the time of blood sample. Similar to Q151M, the prevalence of the mutation was consistently very low in ART-naïve patients, with a maximum of 0.15% (2/1311) in 2003, whilst in ART-experienced patients there was a large fall in prevalence from 0.8% (16/1966) in 2002 to 0.1% (4/3376) in 2006 and then to 0.03% (1/3734) in 2014.

Table 2 provides a summary of the associated major reverse transcriptase mutations at first observation of the T69i mutation in each of the affected patients. Thymidine analogue mutations (TAMs) were most frequently seen, with TAM1 mutations (M41L, L210W and T215Y) being most common. In combination with the T69i, these mutations have been linked to high level resistance to all NRTIs [12]; 63/85 (74.1%) of the patients were observed to have at least one of these mutations present.

**Table 2:** Summary table for associated mutations for T69i (*n *= 85 patients)

	*n* (%)
T69i isolated major RT mutation	2 (2)
T69i associated mutations^a^	
0	16 (19)
1	14 (16)
2	33 (39)
3	22 (26)
NRTI major mutations other than T69i	
0	2 (2)
1	8 (9)
2	19 (22)
3	36 (42)
4	15 (18)
5	2 (2)
6	3 (4)
TAMs present^b^	
0	4 (5)
1	8 (9)
2	37 (44)
3	29 (34)
4	6 (7)
5	1 (1)
NNRTI major mutations	
0	34 (40)
1	10 (12)
2	26 (31)
3	13 (15)
4	2 (2)
PI major mutations	
0	45 (53)
1	10 (12)
2	16 (19)
3	9 (11)
4	4 (45)
5	0 (0)
6	1 (1)
Number of classes with resistance^c^	
1	20 (24)
2	39 (46)
3	26 (31)

^a^TAMs at codons 41, 210 or 215

^b^M41L, D67N, K70R, L210W, T215Y/F and K219Q/E

^c^Of NRTI, NNRTI and PI. A full list of mutations is provided in Additional file 1

**Fig. 4:**
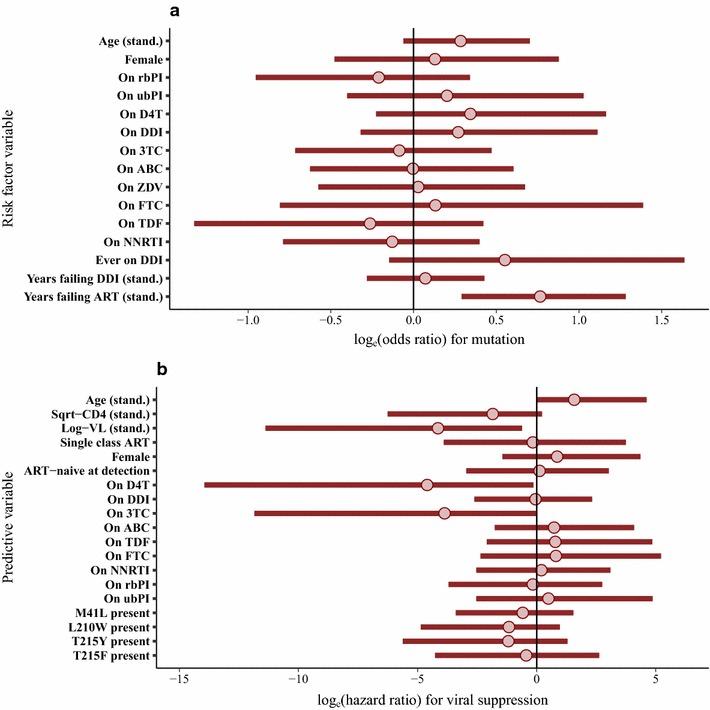
Posterior mean values and 95% credibility intervals for (**a**) log-odds ratios in the matched case–control analysis investigating factors associated with the occurrence of the T69 insertion mutation and (**b**) log-hazard ratios in the Cox regression for confirmed viral suppression following treatment change after detection of T69 insertion mutation. Continuous variables were standardised (stand.), by subtracting the mean and dividing by SD, for these analyses. The results presented are from multivariable models in each case

Of the patients with T69i mutation detected, 45 could be linked to clinical data. Of these patients, 39 (86.7%) were recorded as ART-experienced and six as naïve. Full information regarding the ART regimen prior to the resistance test was available in 36/39 (92.3%) of the ART-experienced patients, and these 36 patients were included in the matched case–control analysis to investigate factors associated with the occurrence of the mutation. For the final fitted model, only ‘total years of virological failure’ showed a substantial positive association with the occurrence of the T69i mutation (standardised odds ratio (sOR), 95% CrI 2.16, 1.34–3.60; OR of 3.48 on original scale (years)), although there was also some evidence for a positive association with ‘DDI use ever’ (OR 1.73, 0.86–5.16) (Fig. 4a). There was no strong evidence of any positive or negative association with viral subtype (results in Additional file 1).

In 33/45 (73.3%) patients, at least one confirmed undetectable VL was observed (at any point) following the detection of the T69i mutation at a median of 1.5 (IQR 0.5–2.2) years from the date of the resistance test blood sample. For a total of 31 patients there were data on change to ART regimen after detection of the T69i mutation and before viral re-suppression, but five of these patients were missing both baseline CD4 cell count and VL (prior to new ART regimen) and so were dropped from the Cox analysis. Hence, 26 patients were included, with a total of 12 events (confirmed viral suppression) observed before censoring. Higher baseline log_10_(VL) showed a substantial negative association with the probability of viral suppression (sHR 0.016, 0.00–0.54; HR of 0.024 on original scale of log_10_ copies/mL), as did the use of D4T (HR 0.010, 0.00–0.88). 3TC (HR 0.021, 0.00–1.02) also showed some evidence of a negative association (Fig. 4b), possibly attributable to the inclusion of two patients on 3TC monotherapy. Very wide credibility intervals were observed for the HR values for several drugs, reflecting the limited data available. There was no strong evidence that linked TAMs were associated with the probability of viral suppression, although negative effects cannot be ruled out, and there were no cases with a K65R mutation included in this analysis. There is some evidence that single amino acid insertions may be associated with a lower level of NRTI resistance [32] and so a sensitivity analysis was conducted only considering multiple amino acid insertions; the results obtained did not show any substantial differences in comparison to the main analysis (details in Additional file 1).

**Fig. 5:**
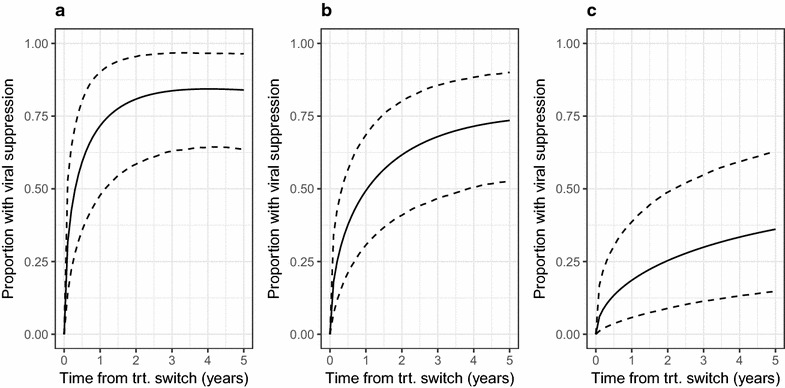
Modelled probability of viral suppression in ART-experienced patients in terms of time since treatment switch following detection of the T69i mutation for patients with a baseline viral load of (**a**) 2000 copies/mL (≈ 10^th^ centile), (**b**) 10,000 copies/mL (≈ 50th centile) or (**c**) 225,000 copies/mL (≈ 90th centile). The expected probability (solid line) and 95% credibility interval (dashed lines) from Bayesian fitting of sequential Weibull models for viral suppression and rebound are shown. D4T use was adjusted for in this analysis, but results are shown for patients not on D4T

A model was fitted to assess the durability of viral suppression that included parameters for baseline VL and D4T use. This analysis indicated that the durability of viral suppression was good for patients with low baseline VL, but that the overall probability of suppression was worse for patients with higher baseline VL values (Fig. 5). The wide 95% credibility intervals for the modelled probability of viral suppression in each plot reflect the small sample size for this analysis.

For the matched cohort analysis of mortality, 35 patients with and 294 patients without the T69i mutation were included. Lower CD4 count was strongly associated with mortality, but no clear association with the number of reverse transcriptase resistance mutations was observed. There was no evidence that presence of the T69i mutation was associated with increased mortality (HR 1.12, 95% CrI 0.64–2.22). In affected patients, the hazard of death was notably higher amongst those with CD4 count < 100 cells/µL (2 deaths/3 patients vs 3/34 for patients with CD4 ≥ 100 cells/µL, further details in Additional file 1).

## Discussion

We have found that the prevalence of multi-drug resistance mutations Q151M and T69i has declined to almost zero in the UK and, as such, it is likely that observation of these mutations will now be very rare in any high-income country. However, the more recent introduction of widespread ART in LMICs has been associated with an increase in transmitted drug resistance [33] and whilst it may be expected that the phase-out of older drug regimens [24] and improved access to VL monitoring [11] will lead to an eventual reduction, the overall levels of drug resistance have yet to show such a decline [34]. Our analysis regarding the risk factors and outcomes associated with these mutations may therefore be relevant for drug resistance monitoring and clinical management of any observed cases in such settings.

Marked declines in prevalence were observed for both Q151M and T69i in the period 2003–2006, but even in the years before this these mutations were relatively rare amongst patients failing ART (1.5–1.7% for Q151M and 0.4–1.3% for T69i) and Q151M was even rarer in ART-naïve patients (0–0.5%) whilst T69i was not observed. The levels amongst treatment-experienced patients are similar to those in subtype-B patients in the Swiss HIV Cohort Study, for which Scherrer et al. reported an overall prevalence up to February 2010 of 0.8 and 0.5% for Q151M and T69i, respectively [14]. In the period 2010–2014 the prevalence of Q151M in treatment-experienced patients undergoing viral sequencing stabilised at around 0.1% in the UK cohort, equating to an average of four cases per year, whilst the prevalence of T69i was even lower in this period at 0.04% (an average of 1.5 patients per year).

Patients who received ART in the earlier years of the HIV epidemic in the UK were treated using a wide variety of drug combinations, with changes often implemented as new drugs became available. The very high level of complexity of treatment histories for HIV hampers epidemiological investigation of risk factors for the occurrence of specific resistance mutations, particularly for the analysis of rare mutations such as Q151M and T69i. There are also equivalent issues to consider when analysing response to treatment conditional on patient characteristics and ART regimen. We were concerned that the analysis of a large number of potential risk (or predictive) factors with a small number of cases would lead to a high risk of false-positive results, and so we chose to carry out our analyses using a Bayesian framework that would penalise estimates of effect sizes without substantial evidence in the data; this approach also has the advantage that results regarding estimation of effect sizes can be interpreted in a directly probabilistic manner rather than purely dichotomised into present or absent.

### Q151M

We found strong associations between D4T exposure and emergence of the Q151M mutation, a link previously identified by Nouhin et al. [15] who found the mutation in 14% (47/328) of patients failing a D4T-containing regimen with at least one resistance mutation present. Tang et al. found a prevalence for Q151M of 4.6% in a review of 1825 patients failing D4T-containing first-line ART [22] drawn largely from LMICs, considerably higher than the peak prevalence observed in the UK. The association with D4T was not identified by Scherrer et al. [14], but their cohort included a relatively small sample of 25 cases of the mutation. A secondary analysis of the EARNEST Trial found a much higher prevalence of Q151M amongst subtype-C patients failing first-line ART (11% vs negligible for other non-B subtypes) [23]. However, in their sample D4T use at failure was much higher (66%) in subtype-C patients than in others (7% when pooled), so this seems likely to have driven the observed difference.

D4T has been subject to a global phase-out over recent years, with a drop in total market share for NRTI use in adults from 29% in 2011 to just 0.2% in 2016 [24], and as such it can be expected that the emergence of this mutation will have undergone a corresponding decrease over this period. However, it is possible that the mutation may persist in a substantial number of patients whilst virological monitoring is limited.

The overall level of confirmed viral suppression (62/96:65%) was similar to that reported by Scherrer et al. (14/25:56%) [14]. We observed that rbPI use showed both a strong negative association with the emergence of the Q151M mutation and a strong association with viral suppression following detection of the mutation, suggesting that this may be a particularly effective treatment option for affected patients. The relative success of rbPI regimens despite broad NRTI resistance is consistent with the findings of secondary analyses of the SECOND-LINE [25] and EARNEST [26] trials of patients failing first-line NNRTI + NRTI regimens, in which those with NRTI resistance switched to rbPI + NRTI regimens showed better virological outcomes than those without resistance mutations. The fact that patients without resistance performed worse on second-line therapy in these studies could be the result of worse adherence within this group, but the results nonetheless indicate that rbPI regimens can be very successful despite viral resistance to the NRTI backbone.

We should note that owing to the historic nature of the cases described, no patients were switched to an integrase inhibitor regimen directly following detection of the mutation and so we have not provided any information on the relative efficacy of this drug class. Our models indicate that virological outcomes were substantially better for patients that had lower baseline VL at detection of the mutation, but it may now be possible to achieve better outcomes for patients with high VL using modern ART regimens.

We did not find evidence that presence of the Q151M mutation is associated with increased risk of mortality relative to comparable patients matched on ART initiation and resistance testing dates, but it is not possible to draw strong conclusions from the limited data available. Mortality outcomes will also be dependent on the availability of effective ART options, and rbPI regimens may not always be available in LMICs. Scherrer et al. [14] assessed mortality with matching to control patients with ≥ 3 TAMs, and in a multivariable model found a non-statistically significant but potentially strong association with the hazard of death (HR 7.5, 95% CI 0.9–64.6). In combination, these results are consistent with the Q151M mutation possibly being associated with the risk of death, but there is once again the caveat that the link could disappear with more modern ART regimens.

### T69i

Recent data from LMICs has indicated that T69i remains a rare mutation, for example Tang et al. identified only two cases (0.1%) in a review of 1825 patients failing D4T-containing first-line ART [22] (this study also included a small minority of patients from the USA and Europe) and Villabona-Arenas et al. found four cases (0.3%) amongst 1599 patients failing first-line ART (ZDV/D4T + 3TC + NVP/EFZ) from 10 West and Central African countries [19]. Scherrer et al. found an association between years spent receiving DDI and emergence of the T69i mutation [14]; we also found some evidence for this link, but we only found a definitive association for total years on ART with virological failure. However, it should be noted that Scherrer et al. carried out an analysis matched against patients with ≥ 3 TAMs, whereas we matched on the basis of ART-start date. The rationale for our choice was to allow investigation of factors associated with appearance of the mutation using comparable patients without requiring the controls to have also failed treatment. As has been reported previously, we found that TAM1 mutations were found in most patients with T69i [35].

The baseline VL prior to treatment switch (or initiation in ART-naïve patients) and continued D4T use were the strongest (negative) predictors of subsequent virological suppression, but the small sample size and large number of potentially important factors made it impossible to rule out important associations with other variables including drug choices and associated mutations. The overall level of confirmed viral suppression (33/45:73%) was very similar to that reported by Scherrer et al. (9/13:69%) [14]. As for the Q151M, we should note that improved outcomes may be possible using modern ART regimens. We did not find any evidence that the T69i was associated with increased mortality, consistent with the findings of Scherrer et al. [14], although we should again note that it is not possible to form strong conclusions given the limited data.

## Conclusions

Our data confirm that modern ART and laboratory monitoring have greatly reduced the occurrence of multi-NRTI resistance due to T69i and Q151M in the UK, a change that is very likely to have also occurred in other developed countries. It remains to be seen whether similar progress can be achieved in LMICs. In addition this report demonstrates that ART regimen changes can be successful despite these mutations, although whether outcomes in patients with the poorest prognosis can be improved with more modern ART regimens also remains to be determined.

## Additional file

**Additional file 1.** Appendix containing further details of methods and results.

## Abbreviations

3TC: lamivudine
ABC: abacavir
AIDS: acquired immune deficiency syndrome
ART: antiretroviral therapy
CRF: circulating recombinant form
CrI: credibility interval
D4T: stavudine
DDI: didanosine
FTC: emtricitabine
HIV: human immunodeficiency virus
HR: hazard ratio
LMICs: low- and middle-income countries
NNRTIs: non-nucleoside/nucleotide reverse transcriptase inhibitors
NRTIs: nucleoside/nucleotide reverse transcriptase inhibitors
OR: odds ratio
PI: protease inhibitor
rbPI: ritonavir-boosted PI
sHR: standardised HR
sOR: standardised OR
T69i: T69 insertion
TAMs: thymidine analogue mutations
TDF: tenofovir
ubPI: un-boosted PI
UK CHIC: UK Collaborative HIV Cohort
UK-HDRD: UK HIV Drug Resistance Database
VL: viral load
ZDV: zidovudine
