# Supplementary material for: Risk factors and outcomes for the Q151M and T69 insertion HIV-1 resistance mutations in historic UK data
Source: AIDS Res Ther. 2018 Apr 16;15:11. doi: 10.1186/s12981-018-0198-7 (PMC5902836; doi:10.1186/s12981-018-0198-7)
Supplement: Supplementary file 1 — Additional file 1. Appendix containing further details of methods and results. [file 12981_2018_198_MOESM1_ESM.docx]

**Appendix to:** Risk factors and outcomes for the Q151M and T69 insertion HIV-1 resistance mutations in historic UK data. Oliver T. Stirrup, David T. Dunn, Anna Tostevin, Caroline A. Sabin, Anton Pozniak, David Asboe, Alison Cox, Chloe Orkin, Fabiola Martin, and Patricia Cane on behalf of the UK HIV Drug Resistance Database and the UK Collaborative HIV Cohort.

**Further details of methods**

Data and general approach

Independent Laplace priors were used for model coefficients, a choice that relates to the Lasso estimate for linear regression[1]. The Laplace distribution is sharply peaked at zero, meaning that posterior parameter distributions show considerable shrinkage towards zero unless there is evidence of a substantial association. The scale parameter for the Laplace distribution, controlling the degree of shrinkage, was not fixed but rather tuned to the data with a hyperprior gamma(1,1) distribution.

Risk factors for development of mutations

In the matched case–control analyses, initially dummy variables for individual drugs were included if 10% or more of the case patients were receiving the drug at time of blood sampling (termed ‘current’ drug variables), with PIs grouped into the presence or absence of a ritonavir-boosted PI or an un-boosted PI, and NNRTIs also all grouped. All analyses also included patient age and sex. For those drugs for which the 80% credibility interval of the associated coefficient excluded zero, dummy variables were then also created for each patient relating to whether they had ever been treated with that drug up until the date of blood sampling in the case patient (termed ‘ever’ drug variables). The model obtained was then further extended by including a variable representing summed periods of observed virological failure whilst on treatment after first ART initiation, and variables representing summed periods of virological failure whilst the patient was being treated using each specific drug found to be positively associated with the resistance mutation (based on a posterior probability of a positive coefficient >90% for the ‘current’ and/or ‘ever’ variable); virological failure was considered to have occurred from the point of any observation of detectable viral load until a subsequent undetectable viral load or a change of treatment regimen. Due to the range of assays used in the period considered, viral load measurements <200 copies/mL were considered to be ‘undetectable’.

Factors associated with successful viral suppression

To allow for delays in implementing changes to treatment guided by the results of the prior resistance test, ART regimens implemented for fewer than 4 weeks were not considered, i.e. change to first ART regime lasting ≥4 weeks was taken as the zero time point. Patients were censored from the analysis at further change to ART regimen or at date of last recorded viral load observation. However, if the patient died without change to ART regimen then they were considered to be included in the analysis at all time points (effectively censored at a greater time than any viral suppression events), in order to reflect the fact that this was likely due to ineffective treatment.

Further analyses were conducted to investigate the durability of viral suppression in ART-experienced patients. Models were developed based on the proposal of McKinnon *et al*.[2], with viral suppression and subsequent virological failure modelled using sequential Weibull time-to-event models. Unlike McKinnon *et al*.[2], we assume events (viral suppression and rebound) to have occurred on the exact dates of VL measurements, rather than treating them as unobserved events between measurement dates. In contrast to the analyses of viral suppression alone, in these analyses we did not censor at change to treatment regime; this is in order to include a greater number of viral rebound events. Viral failure is considered to be the first of two consecutive detectable viral load observations ≥200 copies/mL following confirmed suppression; death after observation of viral suppression was also considered to be a viral failure. Once viral rebound has occurred the model does not allow patients to re-enter a state of suppression. Models were fit using Bayesian methods, but only those variables definitively predictive of viral suppression in the previous analyses were included and so weakly informative priors (normal with mean=0 and SD=5) were used rather than Laplace priors. For these models:

*Q151M:* A model was fitted to assess the durability of viral suppression that included parameters for baseline VL, rbPI and DDI use on the log scale for the scale parameter of the Weibull model for viral suppression, and baseline VL alone for the viral rebound Weibull model. It was necessary to drop the ART-related variables for the rebound model to allow stable model-fitting, probably owing to the small number of viral rebound events observed. A total of 17 viral suppression events were observed in the group whose ART regimen after detection of the Q151M mutation did not include rbPI, and amongst these only one had switched to a rbPI-regimen prior to viral suppression.

*T69i:* A model was fitted to assess the durability of viral suppression that included parameters for baseline VL and D4T on the log scale for the scale parameter of the Weibull model for viral suppression, but no predictive variables for the viral rebound Weibull model.

**Further details of results**

*Q151M*

*Matched case–control analysis for risk factors:* When only the ‘current’ drug variables were included in the model, there was evidence that D4T, DDI and ABC were positively associated with the mutation, whilst a negative association was observed for rbPI and 3TC. When the corresponding ‘ever’ variables were added to the model, evidence of a positive association with the mutation was observed for ‘current’ D4T and for D4T and DDI ‘ever’, with negative associations observed for ‘current’ rbPI and 3TC ‘ever’. When variables summarising the history of viremia were added to the model, the strongest evidence for a positive association was for ‘total years of virological failure’ with occurrence of the mutation. There was no evidence of any positive or negative association with viral subtype (Figure S1).

*Viral suppression and mortality:* Information regarding the presence or absence of accessory mutations was available for 46 of the patients included in the analysis of viral suppression on new ART regimen after detection of the Q151M mutation, but no strong evidence of an association with success of viral suppression was found (Figure S2).

An additional analysis of viral suppression was conducted with variables added relating to PI resistance mutations amongst those patients on a rbPI regimen. If more than one PI was included in a regimen (discounting RTV), then the analysis was conducted according to the drug with greatest viral susceptibility. Four patients showed intermediate viral resistance to the PI in their regimen and a further four showed high level resistance (according to Stanford HIV Drug Resistance Database). However, within the fitted model, resistance status showed no evidence of an association with viral suppression and the findings regarding other variables were unchanged (Figure S3).

In 62/96 (65%) patients, a confirmed undetectable viral load (VL) was observed following the detection of the Q151M mutation at a median of 1.0 (IQR 0.5–2.3) years from the date of the resistance test sample. A total of 13 patients in whom no confirmed viral suppression was observed are known to have died, at a median of 1.9 (range 0.7–3.0) years from date of resistance test, whilst seven patients in whom confirmed viral suppression was observed are known to have died at a median of 4.4 (range 3.1–8.9) years.

Of the 13 patients who died before the observation of viral suppression, the cause of death is recorded as ‘HIV-related’ in two, lung carcinoma in one and unknown in the remaining patients. The cause of death is not recorded in the database for any of the patients who died following observation of viral suppression. Patients who died were disproportionately likely to have received an AIDS diagnosis prior to detection of the Q151M mutation: 16/20 (80%) who died vs 34/77 (44%) who survived had a prior AIDS diagnosis recorded.

A plot of effect estimates from the case–control analysis of mortality in relation to detection of the Q151M mutation is presented in Figure S4. Kaplan-Meier curves generated for overall survival from date of resistance test stratified according to baseline CD4 count <100 or ≥100 cells/µL are shown in Figure S5 (including any patient with available CD4 count without 6 months prior to detection of Q151M mutation).

*Associated occurrence of K65R mutation:* Further information is given regarding this mutation as it confers high level resistance to TDF, whilst Q151M confers low or intermediate resistance. The K65R mutation was also present at first detection of the Q151M mutation in 52/180 (29%) cases (Table S1). Amongst the ART-experienced patients with linked treatment histories, this figure was 23/74 (31%) cases; 10 of the 74 patients had ever been on TDF at this point in time, of which eight (80%) also had the K65R mutation.

Of the 23 cases with K65R at first detection of Q151M, nine had at least one prior viral sequence available and of these prior occurrence of K65R had been observed in 6/9 (67%).

Of the 51 cases of Q151M without K65R at first detection, 34 patients have at least one further viral sequence available. There were six patients in whom the K65R mutation appeared following initial detection of Q151M without K65R.

A full analysis of sequential acquisition of mutations is beyond the scope of this article, but these results suggest that the combination of Q151M and K65R mutations could be acquired in either order.

*T69i*

*Matched case–control analysis for risk factors:* When only the ‘current’ drug variables were included in the model, there was evidence of a positive association between DDI use and the occurrence of the T69i mutation. When ‘DDI use ever’ was added to the model, this new variable showed evidence of a positive association with the mutation (with a reduced association for the ‘current DDI use’ variable). Variables for ‘total years of virological failure’ and ‘years of virological failure on DDI’ were then added to the model; the former showed substantial evidence for a positive association with the T69i mutation, the latter did not but there was still some evidence for an association with ‘DDI use ever’. There was no evidence of any positive or negative association with viral subtype (Figure S6).

*Viral suppression and mortality:* In 33/45 (73.3%) patients, at least one confirmed undetectable VL was observed following the detection of the T69i mutation at a median of 1.5 (IQR 0.5–2.2) years from the date of the resistance test blood sample. Patients with a confirmed undetectable VL after the detection of the T69i mutation were followed up for longer than those in whom no subsequent viral suppression is recorded: median (IQR) time to last measured VL 8.7 (4.6–12.5) *vs* 1.8 (0.5–2.8) years.

A total of four patients in whom no confirmed viral suppression was observed are known to have died, at a median of 2.1 (range 0.8–3.3) years from date of resistance test, whilst one patient in whom confirmed viral suppression was observed is known to have died 15.8 years after the resistance test. A plot of effect estimates from the case–control analysis of mortality in relation to detection of the T69i mutation is presented in Figure S7. Kaplan-Meier curves were generated for overall survival from date of resistance test, and with stratification according to baseline CD4 count <100 or ≥100 cells/µL (5 deaths in 37 patients with CD4 count in prior 6 months recorded) (Figure S8). The cause of death is not recorded for any of the five patients.

*Sensitivity analysis including only multiple amino acid insertions:* The analyses of risk factors for mutation T69i and for predictive factors for viral suppression were re-run considering only those patients in whom there was an insertion of two or more amino acids. The former analysis included 27 case patients (*vs* 36 in the main paper), and the latter included 18 patients (*vs* 26 in the main paper). The results obtained are shown in Figure S9 (comparable to Figure 4 in the main paper). No major differences in results are observed for this sensitivity analysis, although comparison is hampered by the fact that the small sample size makes it difficult to draw any strong conclusions for either version of the analysis.

**Table S1** Summary of major reverse transcriptase mutations also present at first observation of the Q151M mutation in each of the affected patients

| **Mutation** | ***n*** | **%** |  | **Mutation (cont.)** | ***n* (cont.)** | **% (cont.)** |
| --- | --- | --- | --- | --- | --- | --- |
| Q151M | 180 | 100 |  | L74V | 11 | 6 |
| M184V | 84 | 47 |  | T215F | 10 | 6 |
| K103N | 63 | 35 |  | Y188L | 9 | 5 |
| K65R | 52 | 29 |  | G190S | 9 | 5 |
| G190A | 49 | 27 |  | V106M | 9 | 5 |
| D67N | 49 | 27 |  | M184I | 8 | 4 |
| Y181C | 48 | 27 |  | Y181I | 7 | 4 |
| K70R | 40 | 22 |  | E138A | 5 | 3 |
| V108I | 32 | 18 |  | P225H | 5 | 3 |
| Y115F | 31 | 17 |  | K70E | 3 | 2 |
| T215Y | 28 | 16 |  | K103S | 3 | 2 |
| M41L | 27 | 15 |  | K101P | 3 | 2 |
| K219E | 27 | 15 |  | E138Q | 2 | 1 |
| K101E | 22 | 12 |  | Y188H | 1 | 1 |
| H221Y | 21 | 12 |  | M230L | 1 | 1 |
| K219Q | 20 | 11 |  | E138G | 1 | 1 |
| L210W | 14 | 8 |  | V106A | 1 | 1 |
| L100I | 12 | 7 |  | Y181V | 1 | 1 |

**Table S2** Summary of major protease mutations also present at first observation of the Q151M mutation in each of the affected patients (protease sequencing failed in one patient)

| **Mutation** | **n** | **%** |
| --- | --- | --- |
| L90M | 41 | 23 |
| I84V | 21 | 12 |
| M46I | 20 | 11 |
| V82A | 20 | 11 |
| Q58E | 9 | 5 |
| G48V | 8 | 4 |
| D30N | 8 | 4 |
| V82T | 5 | 3 |
| L76V | 5 | 3 |
| M46L | 5 | 3 |
| V32I | 4 | 2 |
| I54L | 4 | 2 |
| N88S | 2 | 1 |
| N83D | 2 | 1 |
| I47V | 1 | 1 |
| V82L | 1 | 1 |
| I50L | 1 | 1 |
| V82F | 1 | 1 |

**Table S3** ART regimen prior to first observation of the Q151M mutation in treatment-experienced patients included in the case–control analysis

| **ART regimen** | ***n*** | **ART regimen (cont.)** | ***n* (cont.)** |
| --- | --- | --- | --- |
| 3TC + ABC + D4T | 1 | DLV + D4T + DDI + IDV | 1 |
| 3TC + ABC + D4T + DDI | 1 | EFV + 3TC + ABC + IDV + RTV | 1 |
| 3TC + ABC + DDI + ZDV | 1 | EFV + 3TC + ABC + ZDV | 1 |
| 3TC + ABC + TDF + ZDV | 2 | EFV + 3TC + D4T | 2 |
| 3TC + ABC + ZDV | 3 | EFV + 3TC + TDF + LPV + RTV | 1 |
| 3TC + D4T | 2 | EFV + 3TC + ZDV | 1 |
| 3TC + D4T + IDV | 3 | EFV + ABC + D4T | 1 |
| 3TC + D4T + NFV | 1 | EFV + ABC + D4T + DDI | 2 |
| 3TC + D4T + NFV + RTV | 1 | EFV + ABC + DDI + NFV | 1 |
| 3TC + D4T + SQV | 1 | EFV + D4T + DDI | 3 |
| 3TC + TDF + APV + LPV + RTV | 1 | EFV + D4T + DDI + TDF | 1 |
| 3TC + TDF + LPV + RTV | 1 | EFV + DDI + ZDV | 1 |
| 3TC + ZDV + IDV | 1 | EFV + IDV | 1 |
| 3TC + ZDV + RTV + SQV | 1 | FTC + TDF + LPV + RTV | 1 |
| ABC + D4T + DDI + APV + RTV + SQV | 1 | NVR + 3TC + ABC + D4T | 1 |
| ABC + DDI | 1 | NVR + 3TC + D4T | 1 |
| ABC + DDI + RTV | 1 | NVR + 3TC + D4T + NFV | 1 |
| ABC + DDI + TDF | 1 | NVR + 3TC + ZDV | 1 |
| D4T + DDC + NFV | 1 | NVR + ABC + D4T | 2 |
| D4T + DDC + SQV | 1 | NVR + ABC + D4T + DDI | 1 |
| D4T + DDI | 2 | NVR + D4T + DDI | 12 |
| D4T + DDI + LPV + RTV | 1 | NVR + D4T + LPV | 1 |
| D4T + DDI + NFV | 1 | NVR + DDI + ZDV | 1 |
| D4T + IDV + NFV | 1 | T20 + 3TC + ABC + DDI + TDF + LPV + RTV + TPV | 1 |
| DDI + ZDV | 1 | TDF + FOS + LPV + RTV | 1 |
| DDI + ZDV + NFV | 1 | Total | 74 |

**Table S4** New ART regimen following observation of the Q151M mutation in patients included in the analysis of subsequent viral suppression

| **ART regimen** | ***n*** | **ART regimen (cont.)** | ***n* (cont.)** |
| --- | --- | --- | --- |
| 3TC + ABC + TDF + LPV + RTV | 1 | EFV + 3TC + ZDV | 1 |
| 3TC + ABC + TDF + ZDV + LPV + RTV | 1 | EFV + ABC + DDI | 1 |
| 3TC + ABC + ZDV + LPV + RTV | 1 | EFV + D4T + DDI | 1 |
| 3TC + D4T + DDI | 1 | EFV + D4T + DDI + LPV + RTV | 1 |
| 3TC + D4T + IDV + NFV | 1 | EFV + D4T + DDI + NFV | 1 |
| 3TC + D4T + IDV + RTV | 1 | EFV + RTV + SQV | 2 |
| 3TC + D4T + NFV | 1 | EFV + TDF + ATA | 1 |
| 3TC + D4T + RTV + SQV | 2 | ETV + DDI + TDF + LPV + RTV | 1 |
| 3TC + DDC + ZDV + APV + LPV + RTV + SQV | 1 | ETV + FTC + TDF + DRV + RTV | 1 |
| 3TC + DDI + TDF + IL2 + APV + LPV + RTV | 1 | FTC + TDF + DRV + RTV | 1 |
| 3TC + DDI + ZDV + NFV | 1 | FTC + TDF + FOS + RTV | 1 |
| 3TC + LPV + RTV + SQV | 1 | LPV + RTV | 1 |
| 3TC + NFV | 1 | MAR + ATA + RTV | 1 |
| 3TC + TDF + LPV + RTV | 1 | NVR + 3TC + D4T + NFV | 1 |
| 3TC + TDF + LPV + RTV + SQV | 1 | NVR + 3TC + ZDV + LPV + RTV | 1 |
| 3TC + TDF + RTV + SQV | 1 | NVR + ABC + DDI + ZDV | 1 |
| ABC + D4T + DDI + IDV | 1 | NVR + APV + LPV | 1 |
| ABC + DDI | 1 | NVR + D4T + DDI | 1 |
| ABC + DDI + NFV | 1 | NVR + D4T + IDV + RTV | 1 |
| APV + FOS + LPV + RTV | 1 | NVR + D4T + LPV + SQV | 1 |
| D4T + DDI | 1 | NVR + DDI + ZDV | 2 |
| D4T + DDI + RTV + SQV | 1 | NVR + TDF + LPV + SQV | 1 |
| D4T + LPV + RTV | 1 | RAL + FTC + TDF + DRV + RTV | 1 |
| DDI + TDF + LPV + RTV | 1 | RTV + SQV | 1 |
| DDI + ZDV + NFV | 1 | T20 + 3TC + DDI + TDF | 1 |
| DLV + D4T + IDV | 1 | T20 + 3TC + LPV + RTV + SQV | 1 |
| EFV + 3TC + ABC + D4T + DDI | 1 | T20 + ETV + 3TC + FTC + TDF + DRV + LPV + RTV | 1 |
| EFV + 3TC + ABC + D4T + DDI + IDV + RTV | 1 | TDF + LPV + RTV + SQV | 3 |
| EFV + 3TC + ABC + IDV + RTV | 1 | Total | 62 |

**Table S5** Summary of major reverse transcriptase mutations also present at first observation of the T69i mutation in each of the affected patients

| **Mutation** | ***n*** | **%** |  | **Mutation (cont.)** | ***n* (cont.)** | **% (cont.)** |
| --- | --- | --- | --- | --- | --- | --- |
| T69Ins | 85 | 100.0 |  | Y188L | 5 | 5.9 |
| T215Y | 54 | 63.5 |  | V108I | 5 | 5.9 |
| M41L | 50 | 58.8 |  | V106A | 4 | 4.7 |
| L210W | 31 | 36.5 |  | L100I | 4 | 4.7 |
| M184V | 29 | 34.1 |  | V106M | 4 | 4.7 |
| Y181C | 23 | 27.1 |  | P225H | 3 | 3.5 |
| K103N | 22 | 25.9 |  | Y115F | 2 | 2.4 |
| K70R | 22 | 25.9 |  | M230L | 2 | 2.4 |
| K219Q | 18 | 21.2 |  | Y188H | 1 | 1.2 |
| G190A | 17 | 20.0 |  | Y181I | 1 | 1.2 |
| T215F | 12 | 14.1 |  | K103S | 1 | 1.2 |
| D67N | 11 | 12.9 |  | G190S | 1 | 1.2 |
| L74V | 10 | 11.8 |  | K101P | 1 | 1.2 |
| K101E | 7 | 8.2 |  | E138A | 1 | 1.2 |
| H221Y | 7 | 8.2 |  | M230I | 1 | 1.2 |
| M184I | 5 | 5.9 |  | Y188C | 1 | 1.2 |

**Table S6** Summary of major protease mutations also present at first observation of the T69i mutation in each of the affected patients

| **Mutation** | **n** | **%** |
| --- | --- | --- |
| L90M | 21 | 25 |
| M46I | 17 | 20 |
| V82A | 16 | 19 |
| V82T | 7 | 8 |
| D30N | 4 | 5 |
| Q58E | 4 | 5 |
| V32I | 3 | 4 |
| I47V | 3 | 4 |
| I84V | 3 | 4 |
| N83D | 3 | 4 |
| L76V | 3 | 4 |
| G48V | 3 | 4 |
| V82F | 2 | 2 |
| M46L | 2 | 2 |
| I54M | 2 | 2 |
| N88S | 1 | 1 |
| I54L | 1 | 1 |

**Table S7** Summary of observed substitutions and insertions (listed following underscore) at 69 position for patients with full sequence available (*n*=67).

| **Mutation** | ***n*** |
| --- | --- |
| T69A_VA | 1 |
| T69A_XG | 1 |
| T69E_T | 1 |
| T69K_T | 1 |
| T69Q_Y | 1 |
| T69S_CA | 1 |
| T69S_CG | 3 |
| T69S_D | 1 |
| T69S_EA | 1 |
| T69S_ES | 3 |
| T69S_LA | 1 |
| T69S_MA | 1 |
| T69S_N | 2 |
| T69S_QG | 1 |
| T69S_SA | 2 |
| T69S_SG | 10 |
| T69S_SN | 1 |
| T69S_SS | 12 |
| T69S_SX | 2 |
| T69S_T | 1 |
| T69S_TA | 1 |
| T69S_TT | 2 |
| T69S_VA | 1 |
| T69S_VG | 1 |
| T69S_VT | 3 |
| T69S_XT | 1 |
| T69S_XX | 1 |
| T69T_I | 1 |
| T69T_N | 2 |
| T69T_PQSP | 1 |
| T69T_T | 6 |
| Total | 67 |

**Table S8** ART regimen prior to first observation of the T69 insertion mutation in treatment-experienced patients included in the case–control analysis

| **ART regimen** | ***n*** |
| --- | --- |
| 3TC + ABC + D4T + DDI | 1 |
| 3TC + ABC + RTV + SQV | 1 |
| 3TC + ABC + TDF + ZDV + RTV + TPV | 1 |
| 3TC + ABC + ZDV | 2 |
| 3TC + D4T | 1 |
| 3TC + D4T + DDI + RTV + SQV | 1 |
| 3TC + D4T + FOS + RTV | 1 |
| 3TC + D4T + IDV | 2 |
| 3TC + D4T + NFV | 1 |
| 3TC + DDI + ZDV + IDV | 1 |
| 3TC + ZDV + IDV | 1 |
| ABC + DDI + TDF + ATA + RTV | 1 |
| D4T | 1 |
| D4T + DDI | 1 |
| D4T + DDI + IDV | 1 |
| D4T + DDI + IDV + RTV | 1 |
| D4T + DDI + RTV | 1 |
| D4T + DDI + RTV + SQV | 1 |
| DDI + ZDV | 2 |
| EFV + 3TC + ABC + D4T | 1 |
| EFV + 3TC + DDI + IDV + RTV | 1 |
| EFV + 3TC + ZDV | 1 |
| EFV + D4T + DDI | 2 |
| EFV + D4T + DDI + NFV | 1 |
| FTC + TDF + FOS + RTV | 1 |
| NVR + ABC + D4T + ZDV | 1 |
| NVR + D4T + DDC + LPV + RTV | 1 |
| NVR + D4T + DDI | 2 |
| NVR + DDI + SQV | 1 |
| NVR + DDI + ZDV | 1 |
| T20 + 3TC + TDF + APV + RTV + SQV | 1 |
| Total | 36 |

**Table S9** New ART regimen following observation of the T69 insertion mutation in patients included in the analysis of subsequent viral suppression

| **ART regimen** | ***n*** |
| --- | --- |
| 3TC | 2 |
| 3TC + ABC + DDI + LPV + RTV | 1 |
| 3TC + ABC + DDI + TDF + ATA + RTV | 1 |
| 3TC + ABC + LPV + RTV | 1 |
| 3TC + D4T + IDV + RTV | 1 |
| 3TC + D4T + NFV | 1 |
| 3TC + DDI + TDF + LPV + RTV | 1 |
| 3TC + DDI + ZDV | 1 |
| 3TC + FOS + RTV | 1 |
| D4T + DDI + IDV + RTV | 1 |
| D4T + DDI + NFV | 1 |
| D4T + NFV | 1 |
| EFV + 3TC + ABC + IDV + RTV | 1 |
| EFV + 3TC + IDV + RTV | 1 |
| EFV + 3TC + ZDV | 1 |
| EFV + D4T + DDI + IDV | 1 |
| FTC + TDF + ATA + RTV | 2 |
| FTC + TDF + DRV + RTV | 1 |
| LPV + RTV + SQV | 1 |
| MAR + RAL + FTC + DRV + RTV | 1 |
| NVR + D4T + DDI + IDV | 1 |
| NVR + D4T + RTV + SQV | 1 |
| NVR + LPV + RTV + SQV | 1 |
| T20 + 3TC + DDI + TDF + APV + LPV + RTV | 1 |
| Total | 26 |

**Figure S1** Posterior mean values and 95% credibility intervals for log-odd ratios in the multivariable matched case–control analysis investigating factors associated with the occurrence of the Q151M mutation, with viral subtype included. Continuous variables were standardised (stand.), by subtracting the mean and dividing by SD, for this analysis.

**Figure S2** Posterior mean values and 95% credibility intervals for log-hazard ratios in the multivariable Cox regression for confirmed viral suppression following treatment change after detection of Q151M mutation, with accessory mutations included. Continuous variables were standardised (stand.), by subtracting the mean and dividing by SD, for this analysis.


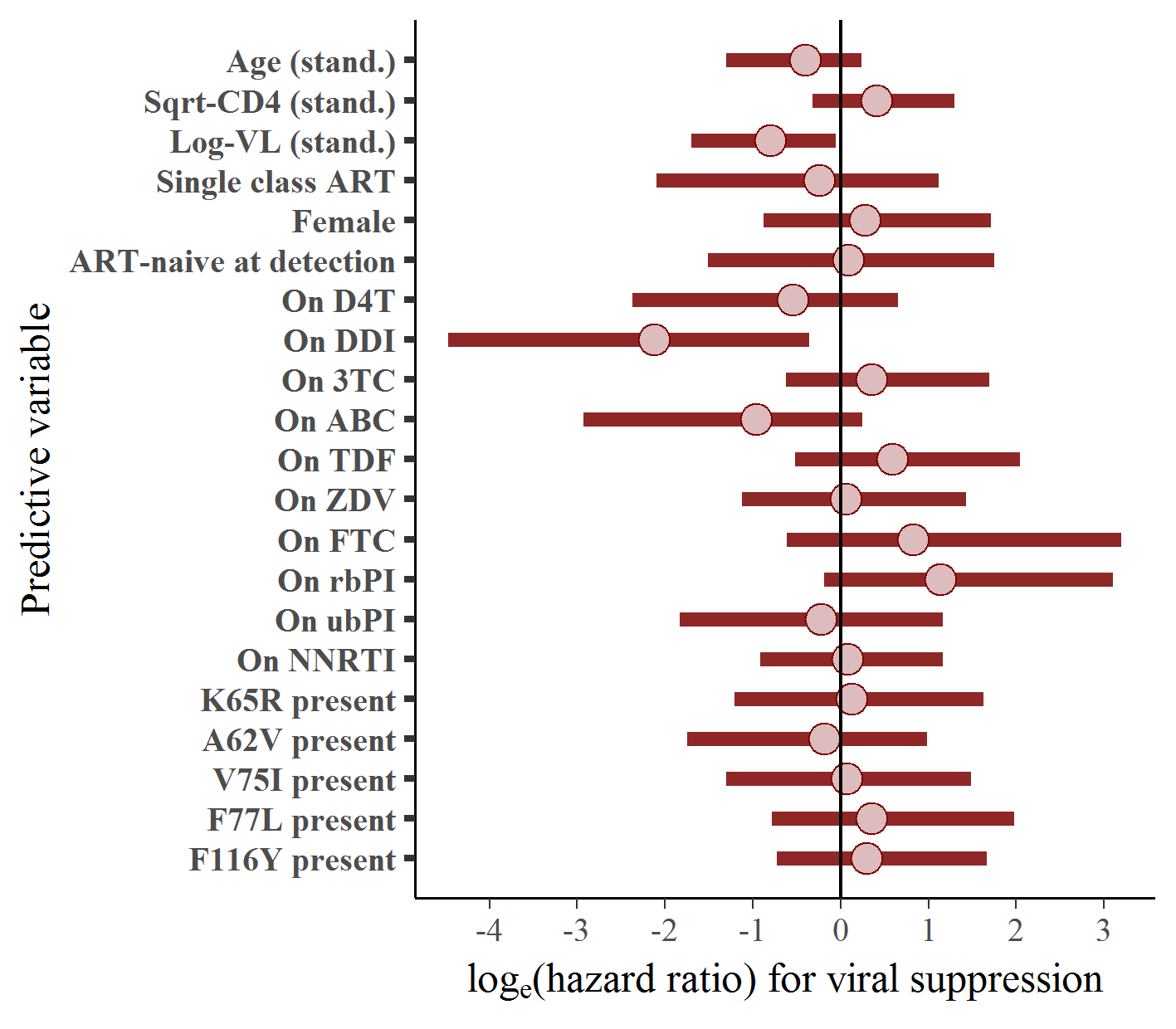


**Figure S3** Posterior mean values and 95% credibility intervals for log-hazard ratios in the multivariable Cox regression for confirmed viral suppression following treatment change after detection of Q151M mutation, with resistance status for those on rbPI regimens included. Continuous variables were standardised (stand.), by subtracting the mean and dividing by SD, for this analysis. PI resistance variables are only included in the model for patients on rbPI regimen.

**
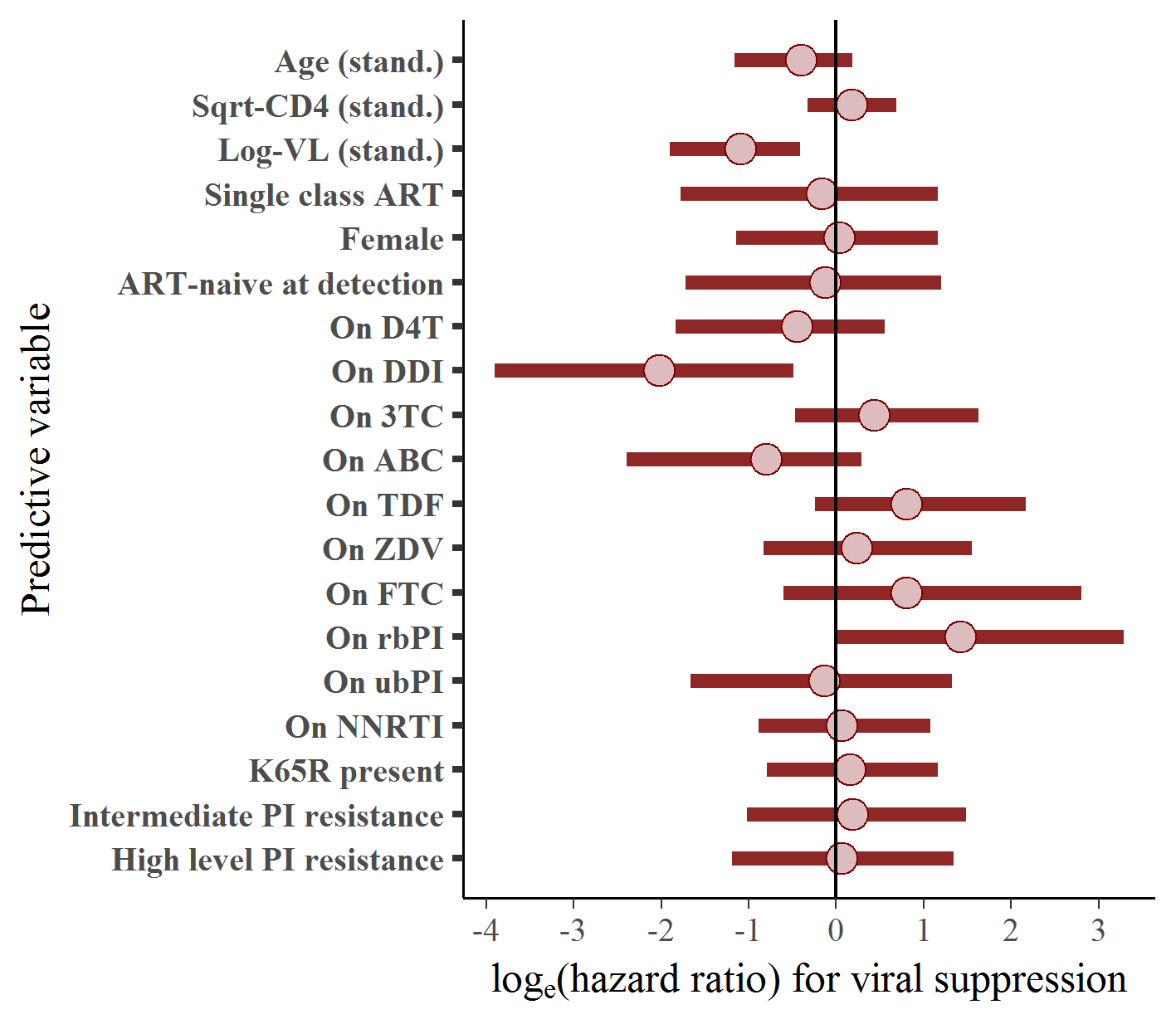
**

**Figure S4** Posterior mean values and 95% credibility intervals for hazard ratios in the multivariable matched cohort Cox regression for mortality after detection of Q151M mutation. Continuous variables were standardised (stand.), by subtracting the mean and dividing by SD, for this analysis.

**
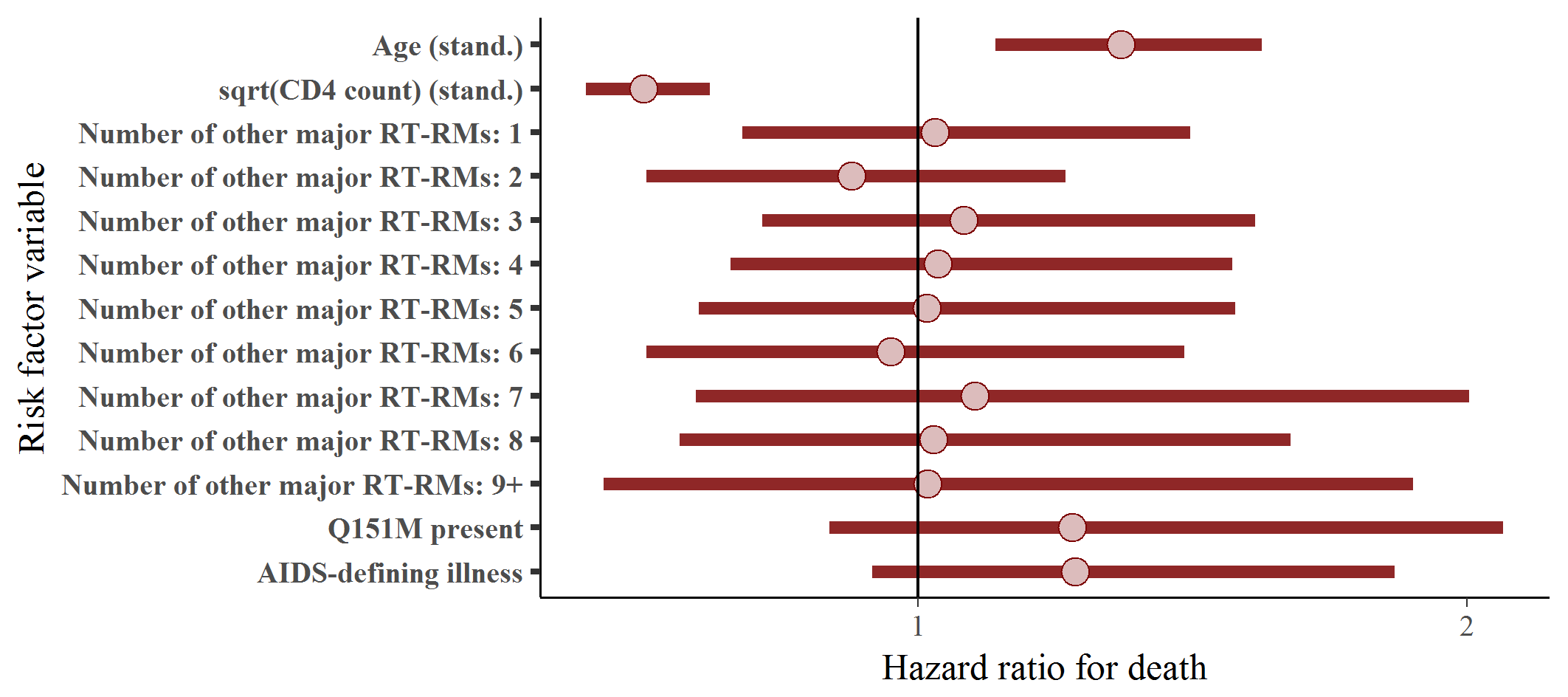
**

**Figure S5** Kaplan–Meier survival graphs (for all-cause mortality) according to baseline CD4 cell count, from date of resistance test showing detection of Q151M mutation.


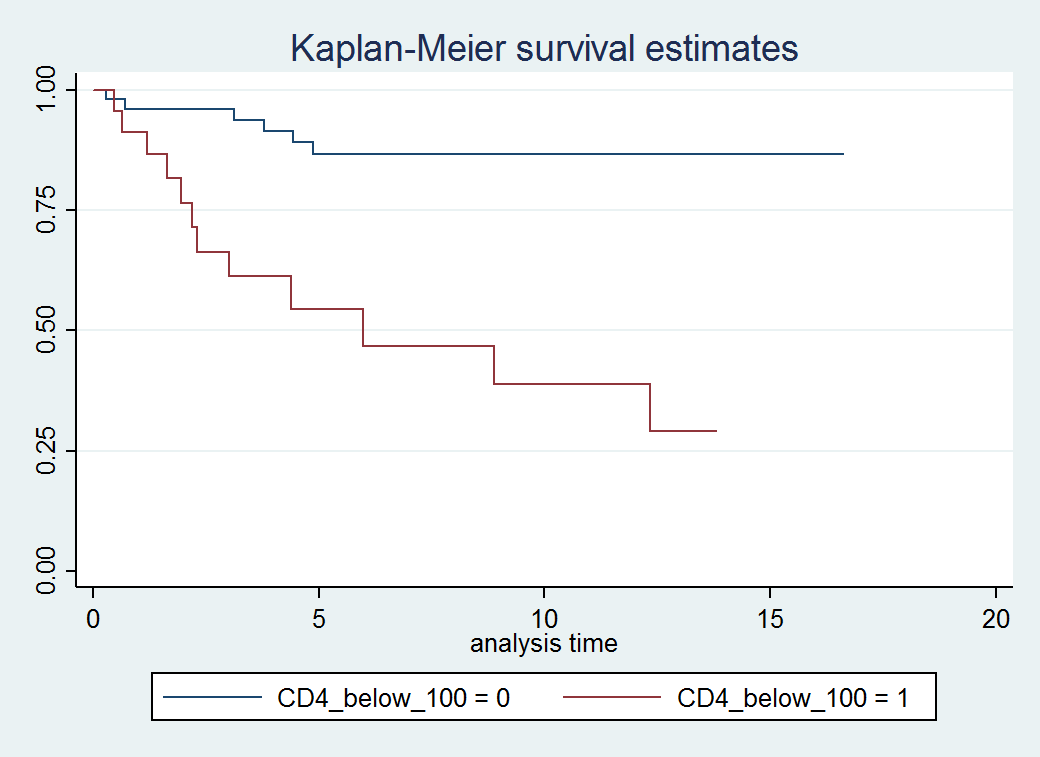


**Figure S6** Posterior mean values and 95% credibility intervals for log-odd ratios in the multivariable matched case–control analysis investigating factors associated with the occurrence of the T69 insertion mutation, with viral subtype included. Continuous variables were standardised (stand.), by subtracting the mean and dividing by SD, for this analysis.

**Figure S7** Posterior mean values and 95% credibility intervals for hazard ratios in the multivariable matched cohort Cox regression for mortality after detection of T69 insertion mutation. Continuous variables were standardised (stand.), by subtracting the mean and dividing by SD, for this analysis.


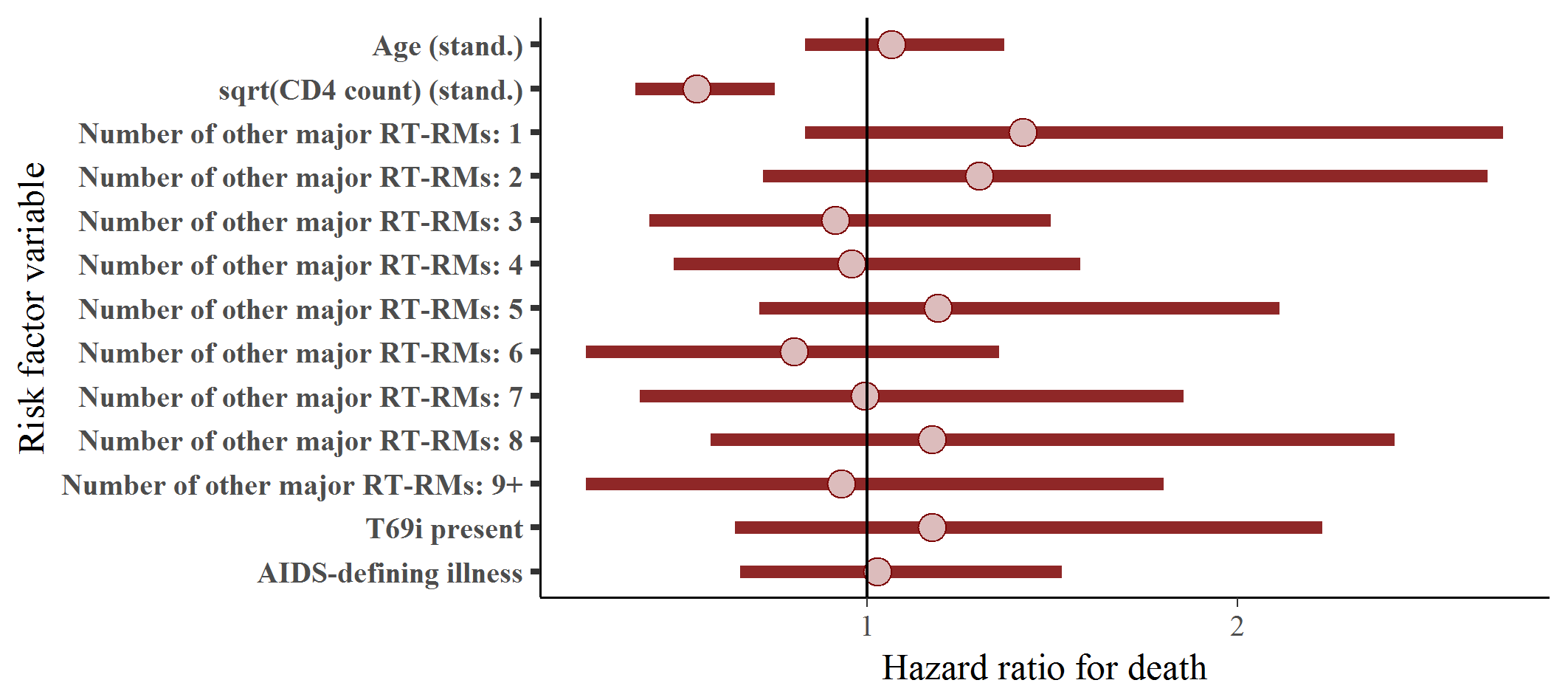


**Figure S8** Kaplan–Meier survival graphs (for all-cause mortality) according to baseline CD4 cell count, from date of resistance test showing detection of T69 insertion mutation.


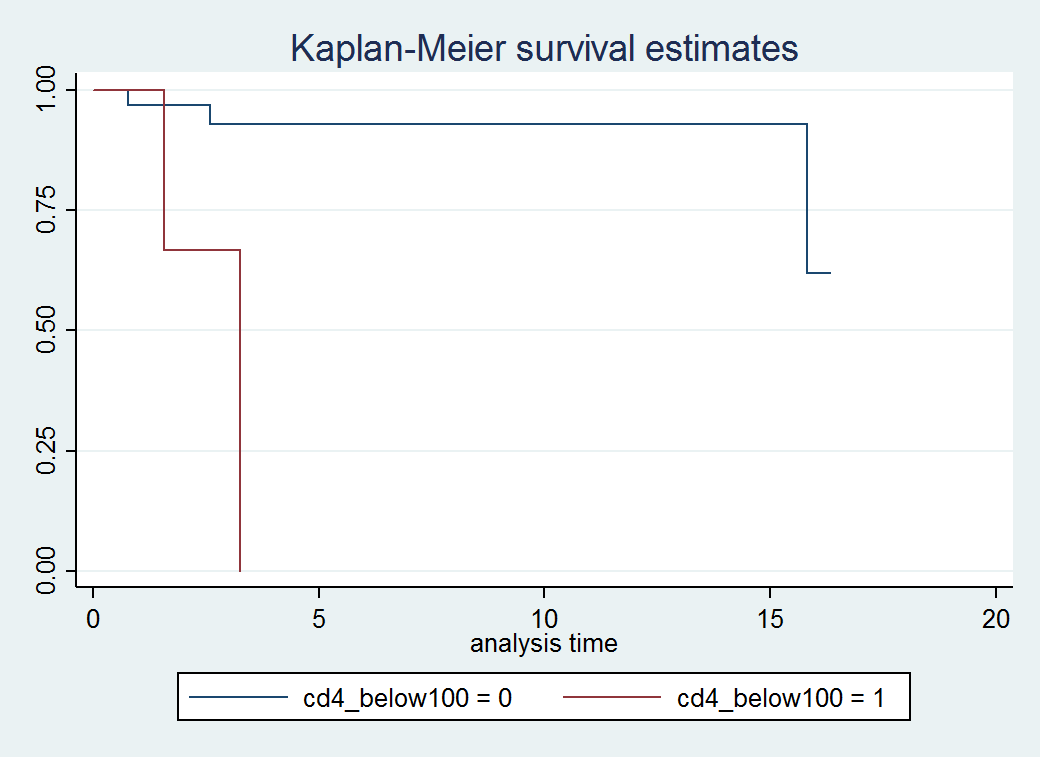


**Figure S9** Sensitivity analysis considering only insertions of two or more amino acids in the definition of T69 insertion. Posterior mean values and 95% credibility intervals for (a) log-odd ratios in the matched case–control analysis investigating factors associated with the occurrence of the T69 insertion mutation and (b) log-hazard ratios in the Cox regression for confirmed viral suppression following treatment change after detection of T69 insertion mutation. Continuous variables were standardised (stand.), by subtracting the mean and dividing by SD, for these analyses. The results presented are from multivariable models in each case.

1. Park T, Casella G. The Bayesian Lasso. *Journal of the American Statistical Association* 2008,**103**:681-686.

2. McKinnon EJ, James IR, John M, Mallal SA. Viral load detectability profiles for HIV infection. *Statistics in Medicine* 2003,**22**:385-396.
